# Potential Predictors of Mortality in Adults with Severe Traumatic Brain Injury

**DOI:** 10.3390/brainsci15091014

**Published:** 2025-09-19

**Authors:** Rachel Marta, Yaroslavska Svitlana, Kreniov Konstiantyn, Mamonowa Maryna, Dobrorodniy Andriy, Oliynyk Oleksandr

**Affiliations:** 1 Department of Allergology and Cystic Fibrosis, Rzeszow University, 35-315 Rzeszów, Poland; mrachel@ur.edu.pl; 2 Department of Anesthesiology and Intensive Care, Bogomolets National Medical University, 01601 Kyiv, Ukraine; kancnmu@nmu.ua (Y.S.); marynamamonova@gmail.com (M.M.); 3 Department of Surgery with a Course in the Basics of Dentistry, Faculty of Postgraduate Education, Vinnytsia National Medical University Named After M. I. Pirogov, Pilotna St. 1, 29000 Khmelnytski, Ukraine; xol.incoming@gmail.com; 4 Department of Anesthesiology and Intensive Care, Ternopil National Medical University, 46000 Ternopil, Ukraine; medicaldepartment@tdmu.edu.ua

**Keywords:** traumatic brain injury, predictors, coagulation, BMI, mortality, machine learning

## Abstract

Background: Severe traumatic brain injury (sTBI) in adults remains a leading cause of mortality and disability worldwide. Early identification of reliable predictors of outcome is crucial for risk stratification and ICU management. Disturbances of hemostasis and metabolic factors such as body mass index (BMI) have been proposed as potential prognostic markers, but evidence remains limited. Methods: We conducted a retrospective, multicenter study including 307 adult patients with sTBI (Glasgow Coma Scale ≤ 8) admitted to three tertiary intensive care units in Ukraine between September 2023 and July 2024. All patients underwent surgical evacuation of hematomas and decompressive craniotomy. Laboratory parameters (APTT, INR, fibrinogen, platelets, D-dimer) were collected within 12 h of admission. BMI was calculated from measured height and weight. Predictive modeling was performed using L1-regularized logistic regression and Random Forest algorithms. Class imbalance was addressed with SMOTE. Model performance was assessed by AUC, accuracy, calibration, and feature importance. Results: The 28-day all-cause mortality was 32.9%. Compared with survivors, non-survivors had significantly lower GCS scores and higher INR, D-dimer, and APTT values. Very high VIF values indicated severe multicollinearity between predictors. Classical logistic regression was not estimable due to perfect separation; therefore, regularized logistic regression and Random Forest were applied. Random Forest demonstrated higher performance (AUC 0.95, accuracy ≈ 90%) than logistic regression (AUC 0.77, accuracy 70.1%), although results must be interpreted cautiously given the small sample size and potential overfitting. Feature importance analysis identified increased BMI, prolonged APTT, and elevated D-dimer as leading predictors of mortality. Sensitivity analysis excluding BMI still yielded strong performance (AUC 0.91), confirming the prognostic value of coagulation markers and GCS. Conclusions: Mortality in adult sTBI patients was strongly associated with impaired hemostasis, obesity, and low neurological status at admission. Machine learning-based modeling demonstrated promising predictive accuracy but is exploratory in nature. Findings should be interpreted with caution due to retrospective design, severe multicollinearity, potential overfitting, and absence of external validation. Larger, prospective, multicenter studies are needed to confirm these results and improve early risk stratification in severe TBI.

## 1. Introduction

Severe traumatic brain injury (sTBI) is one of the leading causes of mortality and long-term disability worldwide. Each year, approximately 70 million people suffer TBI globally, with around 2.5 million cases annually in Europe, of which nearly 1 million require hospitalization and more than 750,000 die [1,2,3]. Mortality rates in patients with sTBI (Glasgow Coma Scale ≤ 8) remain as high as 30–40%, and more than half of survivors experience persistent neurological deficits and reduced quality of life [4,5,6].

Early prognostication of outcomes in sTBI is crucial to guide clinical decision-making, optimize ICU triage, and allocate limited resources. Traditional predictors include demographic characteristics (age, sex), neurological severity at admission (GCS), and systemic complications such as hypotension or hypoxia [7,8,9]. Hemostatic abnormalities are also frequent: up to two-thirds of patients with sTBI develop coagulopathy early after injury, which is independently associated with increased mortality [10,11]. Modern diagnostic approaches, including viscoelastic coagulation tests (ROTEM, TEG, ClotPro), have been proposed as rapid tools for identifying coagulopathy in TBI, offering advantages over conventional plasma-based assays such as PT and APTT [12].

In recent years, attention has turned to metabolic status, particularly body mass index (BMI), as a potential modifier of outcomes in critical illness. Obesity has been associated with prolonged ICU stay, increased complications, and higher mortality in trauma and TBI cohorts [13,14,15]. Therefore, BMI represents a clinically accessible and potentially important prognostic variable.

At the same time, the complex interplay between neurological, hematological, and metabolic parameters suggests that conventional regression models may be insufficient. Machine learning methods, such as Random Forest, can account for nonlinear relationships, high-dimensional interactions, and collinearity among predictors, potentially improving predictive accuracy compared with classical regression [16,17,18].

The aim of this multicenter retrospective study was to identify predictors of 28-day mortality in adult patients with sTBI, with a specific focus on BMI and coagulation abnormalities, and to compare the performance of traditional logistic regression with machine learning-based models.

## 2. Materials and Methods

### 2.1. Study Design and Population

We conducted a retrospective, multicenter observational study including adult patients with severe traumatic brain injury (sTBI), defined as Glasgow Coma Scale (GCS) ≤ 8, who were admitted to three tertiary intensive care units (ICUs) in Ukraine between 1 September 2023, and 30 July 2024. All patients underwent surgical evacuation of hematomas and decompressive craniotomy.

The study was approved by the Biological Ethics Committee of the Ternopil National Medical University (ethics approval number: 2023–145).

### 2.2. Inclusion Criteria

Age ≥ 18 years;
Admission to ICU due to sTBI (GCS ≤ 8);
Surgical treatment for intracranial hematoma with decompressive craniotomy;
Availability of complete laboratory results within 12 h after admission.

### 2.3. Exclusion Criteria

Major extracranial injuries (polytrauma) that could independently affect mortality (e.g., severe thoracic or abdominal trauma);

Presence of comorbidities likely to be the immediate cause of death (cardiogenic pulmonary edema, advanced malignancy, diabetic ketoacidosis, decompensated renal failure, acute myocardial infarction, pulmonary embolism);

### 2.4. Pre-Injury Use of Anticoagulant Therapy

A total of 307 patients met the inclusion criteria; 6 were excluded due to incomplete records, resulting in a final cohort of 301 patients.

### 2.5. Prehospital and ICU Management

Prehospital interventions (airway management, oxygen therapy, fluid resuscitation) were not standardized across sites but generally followed national trauma protocols. The approximate median time from injury to hospital admission was 4 (2–6) h.

In the ICU, management was based on the Brain Trauma Foundation guidelines [19]. The following principles were applied: avoidance of hypotension (mean arterial pressure ≥ 95 mmHg), avoidance of prophylactic hyperventilation (PaCO_2_ 35–44 mmHg), intracranial pressure monitoring (<25 mmHg), osmotherapy (mannitol 0.25–1.0 g/kg), sedation with propofol if required, PaO_2_ > 60 mmHg, and cerebral perfusion pressure around 70 mmHg.

Fluid therapy included Ringer’s lactate and normal saline as standard solutions; hypertonic saline was rarely administered. Nutrition was provided via gastric tube within the first 72 h. Prophylactic enoxaparin was administered from day 4.

### 2.6. Data Collection

Demographic and clinical variables (age, sex, GCS on admission, BMI, time to admission) were extracted from medical records. BMI was calculated as weight (kg) divided by height squared (m^2^). Both weight and height were measured directly at admission; no imputation or estimation was applied.

Coagulation parameters (prothrombin time [PT], activated partial thromboplastin time [APTT], fibrinogen, platelet count, international normalized ratio [INR], and D-dimer) were obtained within 12 h of admission. Abnormal values were defined according to established criteria: INR > 1.2, APTT > 35 s, D-dimer > 800 ng/mL.

### 2.7. Outcomes

The primary endpoint was 28-day all-cause mortality, defined as death from any cause within 28 days of ICU admission. Secondary outcomes (ARDS, multiorgan failure, infections) were recorded but not included as predictors in the present analysis.

### 2.8. Statistical Analysis

Statistical analyses were performed using MedCalc^®^ version 20.009 (MedCalc Software Ltd., Ostend, Belgium). Continuous variables were tested for normality with the Shapiro–Wilk test and reported as median (interquartile range). Categorical variables were expressed as counts and percentages. Group comparisons were made using Mann–Whitney U test (continuous variables) and Fisher’s exact test (categorical variables).

To evaluate predictors of mortality, we used L1-regularized logistic regression and Random Forest classifiers. Multicollinearity was assessed with correlation matrices and variance inflation factors (VIF), with VIF > 10 considered indicative of severe multicollinearity. Due to perfect separation, classical logistic regression could not be estimated. Class imbalance was addressed using Synthetic Minority Oversampling Technique (SMOTE) within training folds during cross-validation. Model performance was evaluated with AUC, accuracy, precision, recall, F1-score, and calibration (Hosmer–Lemeshow test). Feature importance in Random Forest was quantified as relative contribution to prediction accuracy.

## 3. Results

### 3.1. Study Population

Out of 307 screened adult patients with severe TBI, 6 were excluded due to incomplete records. The final cohort included 301 patients: 112 (37.2%) women and 189 (62.8%) men, median age 45 years (IQR 38–54). Median BMI was 29 kg/m^2^ (IQR 28–30), with all patients classified as overweight or obese.

### 3.2. Survivors vs. Non-Survivors

**Figure 1:**
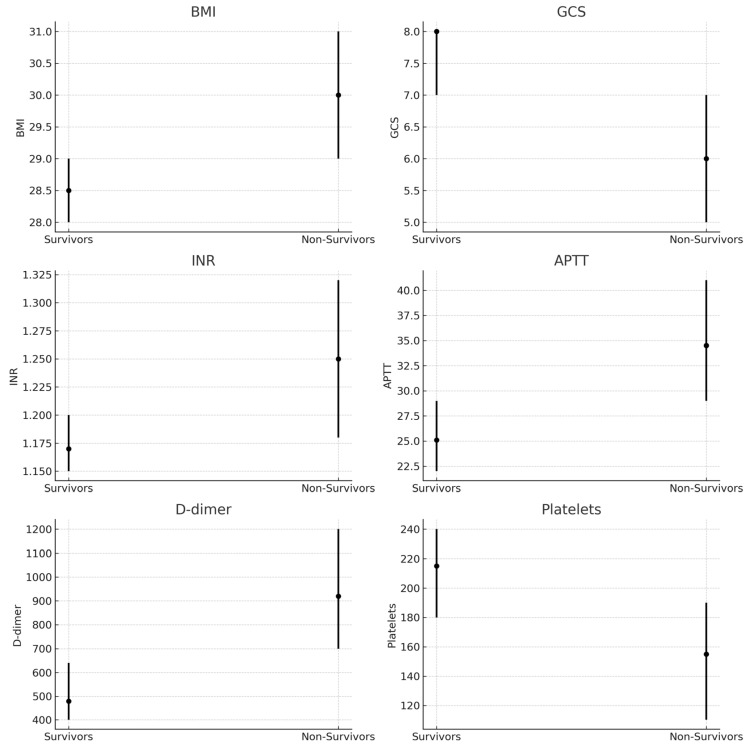
Boxplots of main predictors (BMI, GCS, INR, APTT, D-dimer, Platelets) in survivors vs. non-survivors. Medians and interquartile ranges are shown.

**Figure 2:**
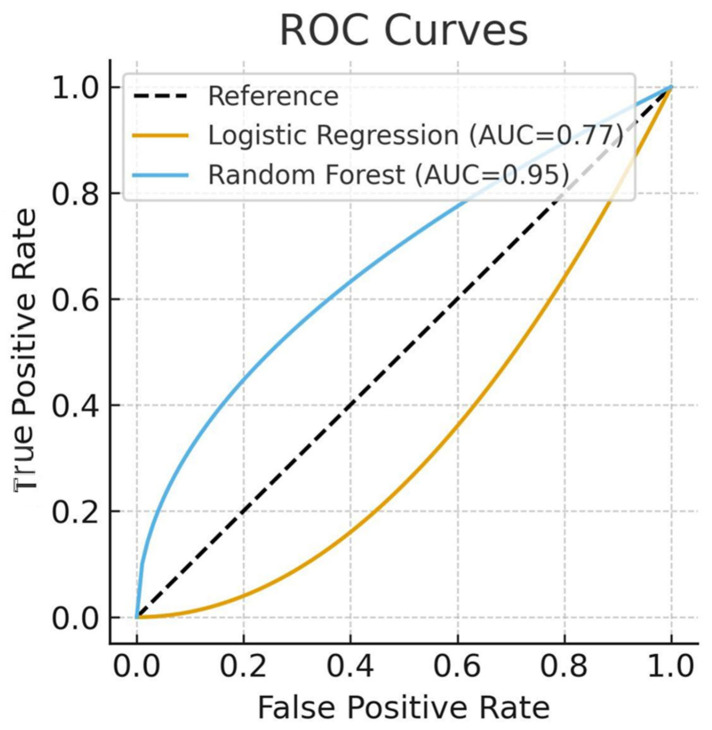
ROC curves comparing logistic regression (AUC 0.77) and Random Forest (AUC 0.95) models.

**Figure 3:**
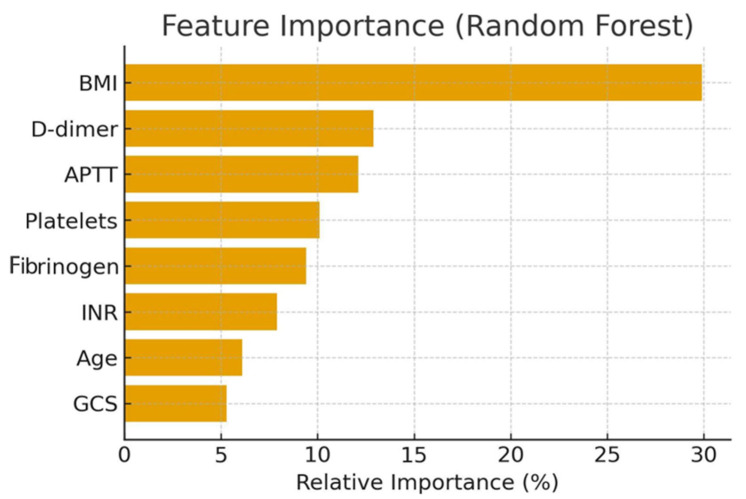
Feature importance plot from the Random Forest model.

**Figure 4:**
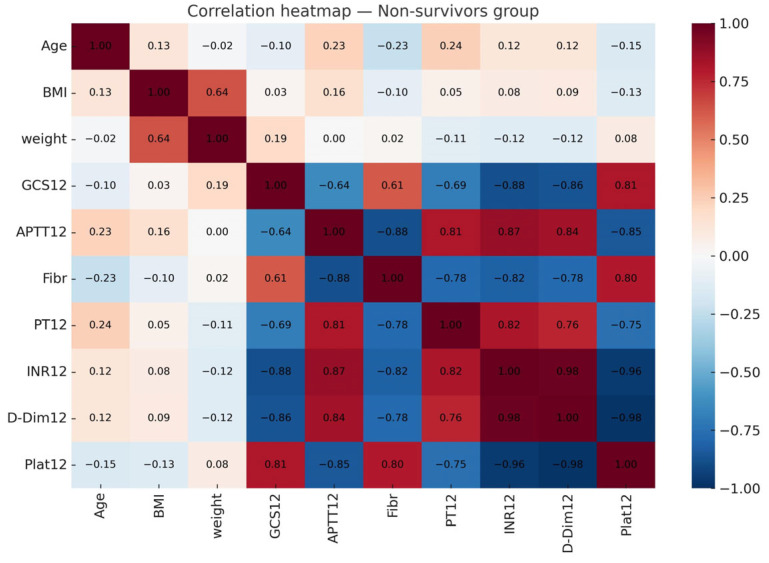
Correlation heatmap of numerical variables in the non-survivors group.

**Figure 5:**
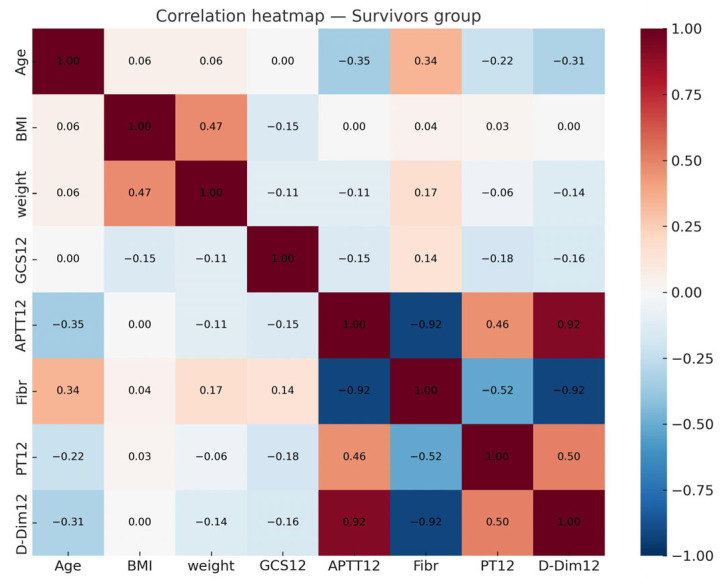
Correlation heatmap of numerical variables in the survivors group.

A total of 101 patients (32.9%) died within 28 days, while 200 (67.1%) survived. Compared with survivors, nrinogenon-survivors had significantly lower GCS scores, higher INR, prolonged APTT, elevated D-dimer, lower platelet counts, and higher BMI (Table 1 and Table 2) (Figure 1, Figure 2, Figure 3, Figure 4 and Figure 5).

Table 1 summarizes key differences between severe TBI survivors and non-survivors, with *p*-values from Mann–Whitney U and Fisher’s exact tests.

### 3.3. Multicollinearity and Regression

Pairwise correlations did not exceed 0.8, yet VIF analysis showed severe multicollinearity (BMI = 507, GCS = 114, INR = 840). This prevented stable estimation with classical logistic regression due to perfect separation. Therefore, we used L1-regularized logistic regression and Random Forest.

### 3.4. Model Performance

Regularized logistic regression achieved AUC 0.77 and accuracy 70.1%, with relatively low sensitivity (recall = 0.40).

Random Forest achieved AUC 0.95 and accuracy ≈ 90%. While performance was high, we emphasized the risk of overfitting given the dataset size, multicollinearity, and SMOTE balancing. Sensitivity analysis excluding BMI yielded AUC 0.91, confirming the prognostic role of coagulation markers and GCS (Table 2).

### 3.5. Feature Importance

In the Random Forest model, the strongest predictors of mortality were as follows:

BMI (29.9%);
D-dimer (12.9%);
APTT (12.1%);
Platelet count, fibrinogen, INR, age, and GCS.

## 4. Discussion

In this multicenter retrospective study of adult patients with severe TBI, we investigated predictors of 28-day all-cause mortality using both traditional logistic regression and machine learning approaches. Our findings highlight the importance of coagulation abnormalities, neurological severity on admission, and BMI as potential prognostic factors. While Random Forest demonstrated high predictive accuracy (AUC 0.95), the results must be interpreted cautiously due to methodological limitations, including multicollinearity, perfect separation, and the small dataset size.

### 4.1. Key Predictors of Mortality

Consistent with prior studies, impaired coagulation parameters (elevated INR, prolonged APTT, increased D-dimer) were strongly associated with poor outcomes [10,11,20]. These markers reflect both consumption of clotting factors and hyperfibrinolysis, processes known to aggravate secondary brain injury. Our analysis also confirmed that lower GCS scores at admission remain one of the strongest established predictors of outcome [7,8,9].

A novel finding in our cohort was the strong influence of BMI, which emerged as the single most important variable in the Random Forest model (29.9% importance). This contrasts with established prognostic models such as CRASH and IMPACT, in which GCS and coagulopathy dominate [21,22]. The narrow BMI distribution (27–33 kg/m^2^) in our sample likely reflects regional population characteristics, with most patients overweight or obese, rather than an artifact of data entry. Nevertheless, this homogeneity may have exaggerated the observed impact of BMI. Importantly, sensitivity analyses excluding BMI showed that coagulation markers and GCS continued to provide strong predictive power (Random Forest AUC 0.91). We therefore caution that BMI’s role as a prognostic factor may be dataset-specific and requires validation in other populations.

### 4.2. Methodological Considerations

Our study faced several important statistical challenges. First, extreme multicollinearity was observed: VIF values for BMI, GCS, and INR exceeded 100, indicating near-perfect redundancy. This explains why classical logistic regression failed due to perfect separation, with certain variable combinations (e.g., very low GCS or highly abnormal INR) almost perfectly distinguishing survivors from non-survivors. Regularized logistic regression and Random Forest were therefore employed as more robust alternatives.

Second, the exceptionally high performance of Random Forest raises concerns regarding overfitting. While nested cross-validation and SMOTE balancing were applied, the small dataset and class imbalance remain limiting factors. The results should therefore be considered exploratory rather than definitive.

Despite these limitations, our findings may have potential clinical utility. Readily available predictors—GCS, BMI, and basic coagulation parameters—could be integrated into early ICU triage and risk stratification algorithms for sTBI patients. Patients with obesity or coagulopathy may represent high-risk subgroups who could benefit from intensified monitoring, early hemostatic interventions, or tailored ICU management. However, these implications remain hypothetical until confirmed in larger and more diverse cohorts.

### 4.3. Comparison with Previous Studies

Our results align with prior evidence linking coagulopathy to poor outcomes in sTBI [10,11,23], while extending this knowledge by exploring the impact of BMI. Previous meta-analyses suggested that obesity may increase mortality in TBI [13,14], although results remain inconsistent. Our findings support further investigation into metabolic factors as modifiers of neurotrauma outcomes. 

Research by [24] found that increased body weight aggravates the course of the disease—OR 1.83 (95% CI: 1.72–1.94), *p* < 0.00001. Among patients with BMI ≥ 30, there are more of those with a GCS score ≤ 8, compared with patients with BMI < 30 with OR 1,08 (95% CI: 1.02–1.14). Length of intensive care unit stay is longer in obese individuals with standard mean difference of 0,29 (95% CI: 0.03–0.55), *p* = 0.03 [24]. BMI ≥ 35 predicts mortality with odds ratio of 3,15 (95% CI [1.06–9.36], *p* = 0.039) [25]. Obesity in patients with severe TBI is associated with longer duration of mechanical ventilation, longer intensive care unit and hospital stay, and, as a result, higher mortality (*p* < 0.001) [17,26].

The theoretical rationale for the influence of the coagulation system on the outcome of TBI is as follows. According to [27], the prevalence of acute coagulopathy is determined by the severity of TBI. Age, pupil reactivity, GCS score, epidural hematoma, and glucose level may act as possible prognostic factors for in-hospital mortality [27]. Almost two-thirds of patients with severe TBI develop hemostatic disorders, which contribute to poor outcome. Coagulation dysfunction in TBI is secondary to tissue injury, leading to coagulation imbalance [28].

According to [13,29], the mortality rate in patients with TBI and coagulopathy ranges from 60.3 to 63.0%.

The generally accepted diagnostic criterion for coagulopathy in severe TBI is an increase in INR over 1.2 [30,31]. Although according to [29], such coagulopathy can also be determined based on an increase in PT ≥ 16.7 s and/or APTT ≥ 28.8 s. There are different opinions regarding the nature of hemostasis disorders in TBI. The first hours are characterized by dysfunction of the coagulation cascade and hyperfibrinolysis, which contribute to the progression of brain tissue damage. This is followed by dysfunction of platelets and a decrease in their number [10]. Secondary hyperfibrinolysis develops as a result of damage to vascular endothelial cells. Increased consumption processes lead to depletion and/or dysfunction of coagulation factors in plasma. It is believed that this occurs due to unregulated release of tissue factor, which stimulates thrombin production [12].

The development of coagulopathy can be predicted by the D-dimer content in the blood [32]. An increase in the D-dimer content indicates an increase in fibrinolysis processes. Fibrinolysis causes an expansion of the hemorrhage zone due to the degradation of coagulation factors and the disintegration of the formed fibrin clot. Dysfunction of the coagulation cascade increases the risk of progression of brain tissue damage [33].

D-dimer peaks several hours after the onset of TBI [34]. Elevated D-dimer levels are associated with an unfavorable neurological prognosis [35]. The likelihood of a fatal outcome increases with D-dimer levels above 1.55 mg/L. In 55% of patients with TBI, D-dimer levels normalize within 3 days [35].

D-dimer studies can be successfully used to diagnose brain damage, since this test is inexpensive and relatively simple [36]. Measuring D-dimer levels can be an alternative to repeat CT [33]. A decrease in platelet count in TBI occurs as a result of increased platelet consumption to form platelet-fibrin clots. The lowest platelet counts are observed 1–5 days after injury, and then they return to baseline levels by days 5–14 [37].

Mortality among patients with TBI and increased INR is significantly higher [38].

According to [13], APTT > 39.2 s can be defined as a discriminator for possible mortality [13]. In this study, the mortality rate of patients with APTT ≤ 39.2 s was 4.5%, and with APTT> 39.2 s—60.8%.

According to [39], with which we agree, the conflicting results of gender-specific differences after traumatic brain injury require more thorough studies using larger study groups.

### 4.4. Limitations

#### 4.4.1. Clinical Implications

This study has several important limitations.

Study design: Retrospective and observational, limiting causal inference.

Population: Single-country (Ukraine), potentially limiting generalizability to other regions and ethnicities.

Sample size: Relatively small for machine learning applications, increasing risk of overfitting.

Multicollinearity: Extreme redundancy between variables complicates regression analysis and interpretation.

Measurement: Laboratory parameters were obtained only once (≤12 h), despite the dynamic nature of TBI-induced coagulopathy.

Missing data: Patients with incomplete records were excluded (n = 6), but no imputation was performed.

BMI distribution: Homogeneous overweight/obese cohort may have biased the observed impact of BMI.

Secondary outcomes: ARDS, infections, and multiorgan failure were recorded but not analyzed as predictors, which may have confounded results.

Clinical practices: Variability in prehospital interventions and fluid resuscitation across centers may have influenced outcomes.

External validation: Absent, limiting generalizability of findings.

#### 4.4.2. Future Directions

Future studies should focus on prospective, multicenter data collection with standardized measurement of predictors, dynamic monitoring of coagulation and systemic inflammation, and incorporation of comorbidities and secondary outcomes. Integration of viscoelastic assays (ROTEM, TEG, ClotPro) and modern biomarkers may further enhance predictive accuracy. Ultimately, externally validated models combining traditional and machine learning methods are needed to support individualized risk stratification in severe TBI.

## 5. Conclusions

In this multicenter retrospective cohort of adult patients with severe traumatic brain injury, 28-day mortality was strongly associated with impaired coagulation parameters (INR, APTT, D-dimer), low neurological status at admission, and elevated BMI. While Random Forest modeling demonstrated promising discriminative performance, the results should be regarded as exploratory given the retrospective design, extreme multicollinearity, class imbalance, and relatively small sample size.

These findings underscore the potential value of simple, routinely available variables—neurological status, BMI, and standard coagulation assays—for early risk stratification in sTBI. However, the strong influence of BMI in our dataset may be population-specific and requires cautious interpretation.

Future work should prioritize prospective, multicenter studies with larger and more heterogeneous cohorts, dynamic monitoring of coagulation, and external validation to confirm the reproducibility and generalizability of these predictors. Only then can such models be integrated into clinical practice for individualized triage and management of patients with severe TBI.

## Figures and Tables

**Table 1:** Comparison of survivors vs non-survivors. (all values median [IQR] or n [%]; *p*-values from Mann–Whitney U or Fisher’s exact test).

Variable	Survivors (n = 200)	Non-Survivors (n = 101)	*p*-Value
Age, years	44 (37–52)	48 (40–56)	0.031
BMI, kg/m^2^	28.5 (28–29)	30.0 (29–31)	<0.001
GCS at admission	8 (7–8)	6 (5–7)	<0.001
INR	1.17 (1.15–1.20)	1.25 (1.18–1.32)	<0.001
APTT, sec	25.1 (22–29)	34.5 (29–41)	<0.001
D-dimer, ng/ml	480 (400–640)	920 (700–1200)	<0.001
Platelets, ×10^9^/L	215 (180–240)	155 (110–190)	<0.001

(legends: INR = international normalized ratio; APTT = activated partial thromboplastin time; GCS = Glasgow Coma Scale).

**Table 2:** Model performance metrics.

Metric	Logistic Regression	Random Forest
AUC	0.77	0.95
Accuracy	70.1%	90.2%
Precision (death class)	0.56	0.88
Recall (death class)	0.40	0.84
F1-score (death class)	0.46	0.86

## Data Availability

The data presented in this study are available on request from the corresponding author. The data are not publicly available due to privacy and ethical restrictions.
